# Observations on the Surgery of the Ancients, Vindicating Their Claims to Many of the Reputed Discoveries and Improvements of Modern Times

**Published:** 1814-08

**Authors:** David Hosack

**Affiliations:** Professor of the Theory and Practice of Physic and Clinical Medicine in the University of the State of New-York, Member of the American Philosophical Society, of the New-York Historical Society, &c.


*For the Medical and Physical Journal.*

### *Observations on the Surgery of the Ancients, vindicating their Claims to many of the reputed Discoveries and Improvements of modern Times.* By David Hosack, M.D. Professor of the Theory and Practice of Physic and Clinical Medicine in the University of the State of New-York, Member of the American Philosophical Society, of the New-York Historical Society, &c.

IN the early ages of society, external accidents constituted the greatest evils to which man was exposed. Intemperance, luxury, and the refinements of civilized life, had not then impaired his bodily constitution, nor debauched his mind. He passed his days in the exercise which was necessary to procure the means of his subsistence, and devoted his nights to rest, undisturbed by care, or those anxieties which at present occupy the civilized portion of the human race.

Man, in a state of nature, was therefore subject to, comparatively, but few diseases; though he was probably not

---

less less exposed to the common casualties of life than at the present day. In climbing the tree to procure its fruits, in traversing the forest, he was necessarily exposed to ordinary accidents; his attention, therefore, would be first directed to the discovery of such remedies as were calculated to remove the evils thus induced. To restore to its socket the dislocated bone, or to replace it when broken; to heal the wounds inflicted by the beast of the field, or the venomous serpent, must necessarily have been among the first objects of his attention. Accordingly, it is observed, by Celsus and other ancient writers, that surgery was cultivated before any other branch of medicine.

The first writers upon physic trace the origin of their art, in common with all other branches of knowledge, to the Egyptians. But the history of surgery, as practised by that people, is so involved in fable, so blended with the pagan mythology, that, notwithstanding the labours of Prosper Alpinus, it is impossible to ascertain their knowledge of medicine, or of any other branch of science. The operations of surgery, stated by the professor of Padua, as having been performed by the Egyptians, namely, the extraction of the stone from the bladder; bleeding in the veins and arteries; the application of the actual cautery, and the paracentesis of the abdomen, in dropsy, must probably have been the operations of the modern and not of the ancient Egyptians.

Let us, therefore, pass over the stories of Hermes and the Egyptian Æsculapius; the fabulous accounts of Osiris, Serapis, Isis, Horus, and Thonis, who were reputed the first practitioners in medicine, and ranked among their divinities, to the more authentic history of the surgery of the Greeks, as related by Homer and Hippocrates.

The first Greek surgeons, on record, are Æsculapius and his sons Machaon and Podalirius. Æsculapius flourished about fifty years before the Trojan war; in which his two sons distinguished themselves, not only by their valour, but by their skill in curing wounds.

In the Iliad, Machaon is spoken of as one of the most distinguished surgeons at the siege of Troy. He is called the *preserver of the Greeks;* and when wounded by Paris, he is lamented, as deprived of the benefit of that skill which he had so often exercised for the benefit of others.

> "The great Machaon, wounded in his tent,
> "Now wants that succour which so oft he lent."*

On

On another occasion, Homer shows the high estimation in which the profession was held:—

> "A wise physician, skilled our wounds to heal,
> "Is more than armies to the public weal." †

Of Podalirius too, it is said, that on his return from the destruction of Troy, he was driven upon the coast of Caria, and that he cured the daughter of Damæthus of a severe and dangerous illness, by bleeding her in both arms. This is the first authentic record of the operation of blood-letting. It is also stated, as an evidence of the value attached to the profession, at that early day, that the prince, as a reward to Podalirius, for his skill and services, gave him his daughter in marriage, with half of his kingdom as her portion.

Notwithstanding these testimonies of the skill of Machaon and Podalirius, it appears that their practice was very much confined to the removal of the darts or arrows with which their wounds were inflicted, and afterwards to the application of fomentations and styptics to the wounded parts; for, when the heroes recorded by Homer were in other respects severely injured, as in the case of Æneas, whose thigh bone was broken by a stone thrown by Diomedes, he makes no mention of any other than supernatural means employed for their relief.

In the writings of Hippocrates, we have a full and circumstantial detail of the state of medicine and surgery among the Greeks in his time. He lived about 400 years before the birth of Christ; and was the first who treated of medicine in a regular or systematic manner. Prior to his time, even among the Greeks, the practice of medicine was confined to their priests and philosophers. According to Celsus, the healing art became united with the duties of religion, from the consideration, that diseases were inflicted upon mankind as punishments for their crimes; and were only to be averted or removed by the intercession of their priests, and the remedies they prescribed. The connection which exists between the study of medicine and the works of nature, also led their philosophers to unite the healing art with their favourite pursuits; and it is related of Pythagoras, that he travelled from place to place, not so much to teach the peculiar doctrines of his philosophy, as to practise physic. Thales, Empedocles, Heraclitus, and Democritus, were among the most distinguished of the Grecian philosophers. They were also celebrated as eminent practitioners of medicine and surgery.

---

Such was the unsettled state of medicine until the time of Hippocrates. In his hands it assumed the form of a distinct science, and was practised as a separate profession. By his labours it became enriched with his valuable and numerous observations on the symptoms, causes, and cure of diseases; and since his time has been respected and cultivated, as among the most important of human pursuits.

Hippocrates not only rescued the profession from the hands of ignorance and superstition, but in his works has left a legacy of great value to the surgeon, as well as to the physician; and, although they are the writings of the most ancient author whose pages are preserved, and written at a period when the structure of the human body, and the functions of the animal economy, were but imperfectly understood; they are the writings of a master, and like the tablets whence he collected many of his observations, they deserve, even at this day, a place in the temple of science, where all can read and profit from the valuable truths they contain.

Among the surgical works of Hippocrates are, a treatise on *Wounds*, a book on *Ulcers*, another on *Fistulas*, a fourth on *Fractures*, and a fifth on *Dislocations*. His observations on abscess, ulcers, and wounds, show him to have been an accurate clinical observer. His work on the joints and dislocations, contains many practical remarks of great value on those subjects. His aphorisms contain principles which are received as so many axioms in physic and surgery; and, at this day, constitute the subjects of academic exercises in the most distinguished universities.

He performed the operation of blood-letting in different parts of the body; and, although he did not understand the circulation of the blood, his observations on the effects of this evacuation, and the diseases in which it is most useful, are judicious and correct. In apoplexy, palsy, inflammatory diseases, iliac passion, quinsy, pleurisy, and inflammation of the abdominal viscera, &c. he made free use of this remedy. In quinsy, and in injuries occasioned by falls, he attached so much importance to blood-letting, that he, in some instances, bled his patient in *both arms*, and frequently *ad deliquum*.

In the treatment of fevers, he was no less cautious in the use of the lancet, than he was bold to employ it in those diseases in which it was decidedly indicated. At the same time that his ignorance of the circulation of the blood occasionally led him into error, the indications of blood-letting, and the circumstances in which it was most advantageously performed, did not escape his notice. The season of the year, the age of the patient, and habit of body, were no less regarded regarded by Hippocrates than they are by physicians and surgeons of the present day. But he was not confined to the use of general blood-letting; he also made use of cupping and scarification for the removal of local diseases.

Hippocrates also performed many other operations, which at this time, notwithstanding the present improved state of anatomy, call for considerable skill and judgment in the operator.

He opened deep-seated abscesses; he performed the operation of tapping in dropsy, and of trepanning in fractures of the skull and injuries of the brain. His aphorism upon the comparative pain and fever, before and after the formation of matter, and which he made the guide of his practice, abundantly shows his accurate knowledge of this subject. His observations on wounds of the head, teach us the great caution with which he formed his prognosis of the event. However small or inconsiderable the injury may appear, he observes, they require attention in their treatment, and care in the practitioner, not to hazard a hasty prognosis of the issue. This observation would do credit to the most enlightened surgeon of modern times; and cannot fail to impress us with an exalted opinion of the talents and experience of Hippocrates.

The *actual cautery* was also one of his favourite remedies, in the treatment of chronic diseases. In a case of ascites, he cauterized the belly eight times, during the forming state of this complaint. The physicians and surgeons of this day have yet to learn many valuable lessons from Hippocrates, and other ancient physicians, relative to the use of this remedy. The successful practice of Pouteau, of Lyons, (see his surgical essays,) who has revived the use of this external application; and the advantages which have resulted from the use of caustics, as prescribed by Mr. Pott, in diseases of the spine, tend to establish the importance of the principle upon which the actual cautery was prescribed, and has been found useful.

Many other local applications were made use of in the practice of Hippocrates, that are still employed by the phycians and surgeons of this day.

In an inflammation of the throat, he caused his patients to inhale the steam arising from the infusion of stimulant and aromatic herbs: upon other occasions, he directed fomentations to be applied to the parts affected. The warm bath, fomentations, gargles, ointments, cataplasms, and collyria, were also among the local remedies prescribed by this celebrated physician.

The important operation of lithotomy appears, also, to

 have have been performed in the time of Hippocrates; for, in the oath administered to his pupils, he exacts from them the obligation, that they *would not cut for the stone.*

Although the operation, as performed by the surgeons of that day, was apparently simple, compared with the present lateral operation; still, from their imperfect knowledge of the structure of the human frame, it must necessarily have been attended with considerable danger. Even, as improved at this time, it is an operation frequently productive of dangerous consequences; and, in some instances, proves fatal in the hands of the most skilful surgeon. The conduct of Hippocrates, therefore, in prohibiting his pupils from performing so important an operation, at the time the anatomy of the body was imperfectly understood, must give us a high opinion of the correctness of his judgment and his prudence, at the same time that it attaches an additional value to his works.

In the interval of time between Hippocrates and Celsus many surgeons are recorded by Galen and other historians; but, as they appear to have made but few and inconsiderable improvements in the art, we will pass over this period to the Augustan age, which may also be denominated the Augustan age of medicine as well as of science. Such it was rendered by Celsus, the Roman Hippocrates.

Celsus was not only distinguished for his professional acquirements, but ranks among the most celebrated writers of antiquity. Whoever, therefore, wishes to become acquainted with the state of medicine and surgery, prior to the fall of the Roman empire, or to read the Latin language, which, in that day, attained its greatest purity and elegance, will peruse his valuable pages, which have gone through almost innumerable editions, and have been translated into almost every language employed as the vehicle of learning. Celsus not only exhibits an historical view of the state of medicine, as practised before his time, but has added much original observation, and many improvements, to those of his predecessors. Although he has copied much from the writings of Hippocrates, he was not so blindly attached to his works as not to discriminate between his merits and his defects. On the contrary, while he gave the opinions and practice of others, he thought for himself. His works are accordingly esteemed the most valuable repository of the medical learning of his time. He treats of the healing art, in all its branches of *diet*, *medicine*, and *surgery*. His seventh and eighth books are exclusively devoted to the latter subject, and contain a systematic view of most of the diseases and operations which fall to the province of the surgeon.

In

In his observations on *wounds*, *abscesses*, and *ulcers*, he describes the various appearances they assume, and adapts his remedies to the different stages, with as much correctness as is done by the surgeons of this day. To wounds, accompanied with hemorrhage, he applied constant pressure, by means of a sponge, wet with vinegar. If necessary, he applied the ligature to the bleeding vessel, or closed its orifice by the actual cautery. When attended with inflammation, he enjoined abstinence upon the patient, and covered the part affected with cold applications. In *contusions*, he freely opened the bruised part, or dilated the wound for the evacuation of the blood effused, when there was no danger of injuring the larger blood-vessels, or important nerves. In gangrene, occurring in the extremities, he cut down to the sound parts; and, if he failed by that and other efforts to conquer the disease, he recommended the amputation of the limb. To promote the suppuration of abscesses, he employed poultices, composed of barley-meal, marsh-mallows, or linseed and fenugreek; but when opened, he prescribes stimulant food and drinks, and to the parts he applied honey and other digestives. When the lips of the wound became callous, its surface spongy and insensible, and the discharge of an unhealthy appearance, he washed the wound and the surrounding parts with *wine*. From this practice of Celsus, the surgeons of the present day may learn an important lesson in the treatment of wounds. He also describes the symptoms of that dangerous abscess the *carbuncle*, and advises the actual cautery to corrode the gangrened part. This practice is now superceded by the internal and external use of the peruvian bark, and other stimulants. In erysipelas, he applied cerussa, with the juice of *solanum*, a powerful sedative. In the treatment of the callous ulcer, he removed the hard edges with the knife, or corrosive applications. In cancers, and in the cancerous ulcer, he prescribed the auripigmentum or arsenic. In fistulous ulcers, if tortuous in their course, with the probe as his guide, he ascertained their direction, divided them freely with the knife; and, upon exposing their internal surface to view, he removed the callous portions, and applied such dressings as were calculated to promote the growth of new parts. In other instances he effected a cure by the use of stimulant and corrosive injections. In caries of the bones, he directs them to be perforated as deep as the disease extends, and afterwards a hot iron to be passed into the foramina, made by the perforator, for the purpose of drying and separating the diseased portions; but if the caries and blackness extend through the body of the bone, he advises a total separation of the part affected.

In

In his chapter on tumours, he described them according to the nature of their contents, and advises their extirpation. The *steatomatous* tumour, from the firmness of its consistence, he directs to be carefully separated from the skin and surrounding parts, and to be removed entire, without dividing the sac inclosing it. He then directs the lips of the wound to be brought together, and an application made to agglutinate the divided parts, at the same time making use of wine and other external stimulants to promote their union. In this treatment, we see the improvements of the present time, of *saving skin*, and *healing by first intention.* That this was the object of Celsus appears evident from a subsequent direction which he gives, in case any portion of the sac should be left behind, as in the removal of the melicerous or atheromatous tumour; in which case, he orders the wound to be left open, and digestives to be applied, for the purpose of throwing off the remaining portion of the sac. In the atheroma of the eye-lids, in which the sac adheres very slightly to the surrounding parts, he directs the teguments to be divided, without wounding the sac, and the tumour to be removed by the fingers, without further dissection; for, he adds, it easily separates. The same mode of treatment is recommended in the work of Mr. Hey, as a valuable improvement of the present time. In his chapter on diseases of the eyes, Celsus treats of the *cataract,* and directs it to be depressed by the needle; and if it rises again, to be broken into pieces; the practice recently adopted by the most eminent surgeons. In *ophthalmia,* he prescribes venesection, purgatives, abstinence, low diet, rest, and a dark room. He washes the eye with collyria, composed of an infusion of roses and the poppy. When attended with defluxion, he employs astringents, cupping, and the actual cautery, to the temple and forehead, analogous in its effects to blisters and issues, as at present prescribed, under similar circumstances.

Celsus also performed the operation of the hare-lip. He extirpated the polypus from the nose; he removed, by excision, the enlarged and indurated tonsils; he diminished the elongated uvula; and he removed the bronchocele, by caustics and the knife. But the skill of Celsus was not confined to the smaller operations of surgery.

In diseases of the bones of the head, and in injuries of the brain, his practice was such as the most eminent surgeons of the present day must approve. The symptoms which are occasioned by a fracture of the skull, are minutely detailed. He is the first who notices the rupture of the vessels of the brain, and the other effects produced by a concussion without fracture of the cranium. He also observes, that the evidence of of such injury, is generally a pain immediately above the part affected; and, upon exposing the bone to view, he adds, it is found of a *pale colour.* This characteristic symptom of effusion of blood, or of the formation of matter between the cranium and the membranes of the brain, is mentioned in the works of Mr. Pott, but without the credit due to that accurate observer. In cases of this sort, and in fractures of the skull, it was the practice of Celsus to make a free crucial incision of the integuments; by which alone, in many instances, the patient was relieved. At the same time, he condemns the precipitate conduct of his predecessors, in proceeding to the use of the trepan, without waiting to observe the effects of other remedies.

(To be continued.)

